# Postoperative pain management in children

**DOI:** 10.3389/fped.2026.1777446

**Published:** 2026-03-20

**Authors:** Pierre Pardessus, Yara Maroun, Lynda Ferahtia, Zied Sammoud, Refka Kaddour, Chiraz El Bachraoui, Myriam Abdelmassih, Joelle Saroufim, Kelly Brouns, Florence Julien-Marsollier, Sonia Benkalifa, Souhayl Dahmani

**Affiliations:** 1 Université de Paris-Cité, Paris, France; 2 Department of Anesthesia and Intensive Care, Robert Debré University Hospital, Paris, France; 3 FHU I2D2, Robert Debré University Hospital, Paris, France; 4 Department of Anesthesia and Intensive Care, Robert Ballanger Hospital, Aulnay-Sous-Bois, France

**Keywords:** pain, pediatrics, persistent pain after surgery, postoperative pain, rehabilitation

## Abstract

Despite considerable research and established guidelines, postoperative pain management in children remains suboptimal. This review aims to outline the significant developments in pediatric postoperative pain management over the past decade and to provide updated, evidence-based recommendations. A comprehensive literature search was conducted across major databases, followed by detailed analysis. In addition to the core strategies of systemic non-opioids, systemic opioids, and regional analgesia, two major developments have significantly affected clinical practice: the widespread implementation of Enhanced Recovery After Surgery (ERAS) protocols and the emerging research on chronic postsurgical pain (CPSP). ERAS protocols have played a crucial role by incorporating minimally invasive surgical techniques, which effectively reduce postoperative pain and accelerate recovery. A fundamental component of ERAS is the intentional minimization of opioid exposure, achieved through standardized opioid-sparing multimodal protocols and the proactive application of regional anesthesia. Concurrently, there is increasing awareness of CPSP as a significant long-term complication. Although its pathophysiology mechanisms remain incompletely understood, but research has shown a strong statistical correlation between CPSP development and both high levels of postoperative pain intensity and increased opioid consumption. This connection guides current preventive strategies, as CPSP affects up to 50% of pediatric surgical patients, with a higher prevalence after major surgeries. Consequently, optimizing acute pain management within an ERAS framework—by reducing both pain and opioid use—is hypothesized to serve as a protective measure against CPSP. Finally, effective postoperative pain management in children requires a multifaceted approach. This includes multidisciplinary collaboration, adherence to standardized, evidence-based protocols, continuous professional education, and individualized patient follow-up.

## Introduction

A decade ago, we published a review on postoperative pain management (1).

Re-examining of that publication and comparing it with the current literature reveals that the majority of pharmacological and technical interventions employed at that time remained largely unchanged. This observation raises an important question: what substantive advancements have occurred over the past ten years? Postoperative pain continues to be a significant cause of patient suffering and a major clinical concern. Earlier research reported that up to 50% of children experienced severe pain following tonsillectomy (2). Recent data indicates that this statistic remained unchanged (3), which is surprising given the large number of publications and clinical guidelines on pain management, especially for procedures like tonsillectomy (4, 5).

Fortunately, significant progress has resulted from concurrent developments including: the adoption of new, less invasive surgical techniques; improvements in pain monitoring; and the widespread implementation of Enhanced Recovery After Surgery (ERAS) protocols. These strategies aim not only to reduce immediate postoperative pain but also to mitigate the risk of persistent or chronic postsurgical pain (6, 7). Although, no relation has been demonstrated between early opioid use and persistent or chronic postsurgical pain, a statistical relationship between these two characteristics has been identified (8). This statistical relationship suggests that a reduction in morphine consumption associated with the implementation of ERAS protocols might prevent the occurrence of persistent or chronic postsurgical pain (6, 7) (refer to the section on Persistent Pain After Surgery and Perspectives in Its Prevention). This review aims to provide clear, updated guidelines for managing postoperative pain in children after surgery. We will outline existing treatment options, advancements in pain monitoring, surgical innovations designed to reduce pain intensity, and key perioperative management strategies. Finally, we will discuss the impact of these treatments in preventing long-term pain.

### Methodology

The present review was conducted through a systematic process comprising the following steps: a literature search and selection, critical analysis of the identified studies, and manuscript preparation based on the outcomes of the literature evaluation.

A comprehensive literature search was performed using the PubMed and Embase databases, employing the following search terms within titles and abstracts in English language: “postoperative pain” and “children,” with filters applied to include only clinical trials and systematic reviews, due to the anticipated volume of results; “nociception” and “children”; and “chronic pain,” “surgery,” and “children.” The most recent search occurred on January 31, 2026.

Articles related to postoperative pain were included in the analysis if they met the following criteria: randomized controlled trials or systematic reviews focusing on the management of postoperative pain in children; articles were excluded if they fell outside the defined scope or were performed for dental, cardiac or neurosurgery. Due to the limited number of controlled trials for the two other searches, articles were included if they were within the scope of the search and excluded if they were not in the defined scope, related to the treatment of chronic pain or were performed for dental, cardiac or neurosurgical procedures.

**Figure 1:**
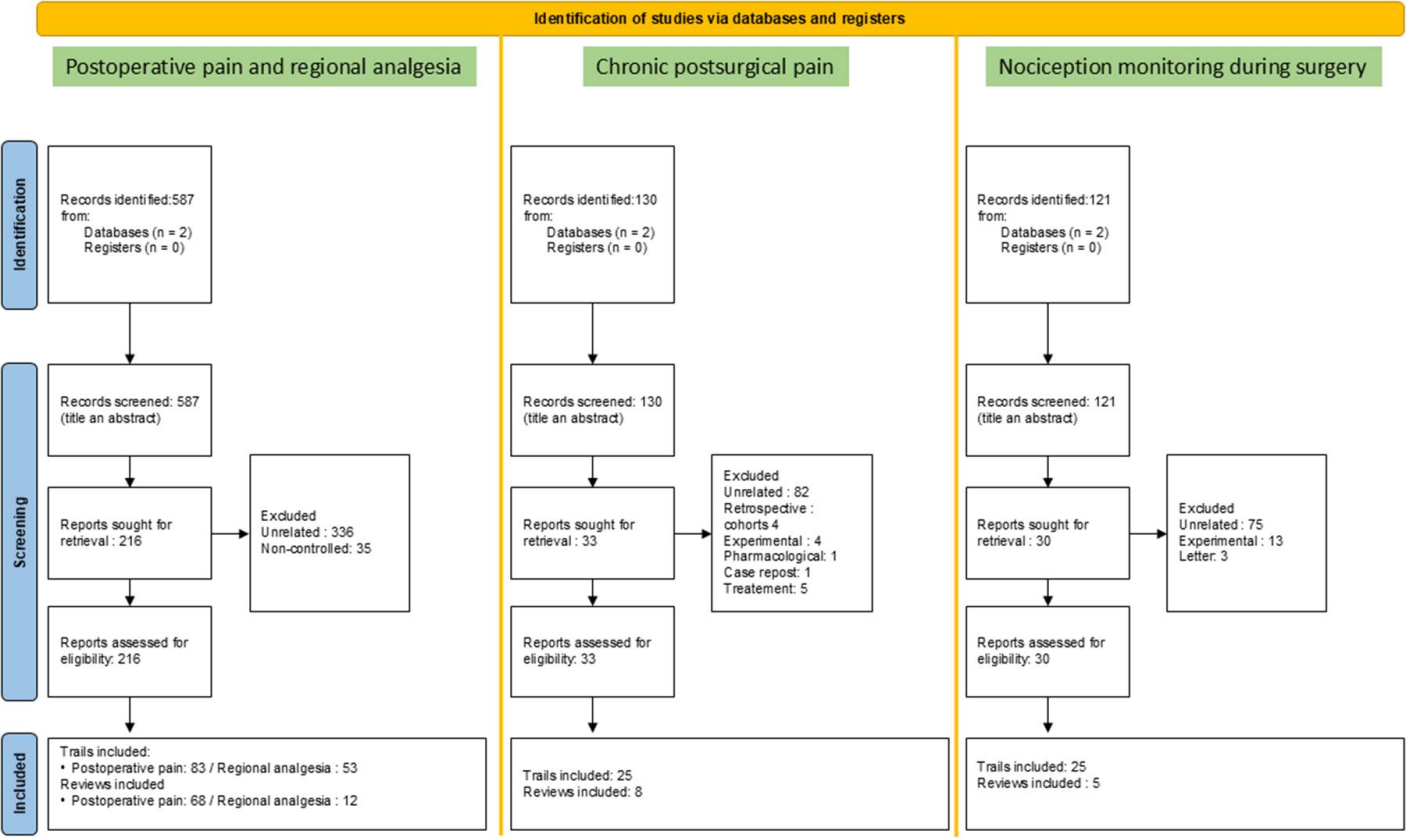
Flowchart of included studies.

The selected articles were read by all co-authors based on the following distribution: SD (chronic pain), FJM (nociception monitoring), PP and YM (regional analgesia), LF ZS, RK, CE, MA, JS, KB and SB (postoperative pain management). Results were summarized prior to the planning of the review, and additional references were added if necessary, depending on the development of the manuscript. Figure 1 illustrates the flowchart of the analyzed studies.

### Pain assessment

Accurate pain assessment is essential for the effective management of postoperative pain. Therapeutic interventions must be evaluated using quantitative measures to guarantee reliable and reproducible results. Pain assessment is founded on two key principles: firstly, self-assessment is considered superior to observer-based evaluation; secondly, a consistent assessment instrument should be employed for each patient throughout the course of their treatment (9–11).

The selection of tool is contingent upon the child's age and cognitive capacity (11, 12). For school-age children, standard self-report tools include the Visual Analog Scale or Faces Pain Scale. In contrast, assessment methods differ for younger children or those with neurocognitive impairments, where observer-based tools are necessary. These tools combine physiological and behavioral variables (e.g., crying, movement, agitation).

The commonly used scales (Table 1) include the FLACC (Face, Legs, Activity, Cry, Consolability) scale (11, 13, 14), CHEOPS (Children's Hospital of Eastern Ontario Pain Scale), OPS (Objective Pain Scale) (12), as well as parental report measures such as the PPPM (Parent's Postoperative Pain Measure) (12). Parental scales are primarily designed for use at home following hospital discharge; however, research indicates that even when parents recognize severe pain, their management is often inadequate.

**Table 1:** Assessment tools for pain monitoring in children and infants.

Acronym	Age range
CHIPPS	Under 5 years
CHEOPS	1–7 years
FLACC	2 months–7 years
OPS and MOPS	8 months–13 years
Poker Chip Tool	From 3 years
Oucher Scale	3–12 years
Wong–Baker FACES® Pain Rating Scale	From 3 years
FPS-R	From 4 years
VAS	From 5 years
NRS	From 8 years

CHIPPS, children and infants postoperative pain scale; CHEOPS, children's hospital of Eastern Ontario pain scale; FLACC, face, legs, activity, cry and consolability; OPS/MOPS, objective pain scale/modified objective pain scale; FPS-R, faces pain scale—revised; VAS, visual analogue scale; NRS, numeric rating scale.

Recently, remote monitoring technologies utilizing smartphone applications or SMS-based communication have been introduced to enhance the quantification of pain after discharge. However, the advantages of these techniques are not yet well-established, which may limit their adoption (15).

Nociceptive monitors, which have been validated in children and infants over the past decade, are widely used in anesthetized patients where behavioral assessment is not feasible (16, 17).

Their potential benefits in conscious patients during the early postoperative recovery phase, although their comparative efficacy relative to traditional scales remains uncertain (18, 19). They could be particularly advantageous for patients with neurocognitive impairments, though this application is not yet well-studied. Alternatively, specific scales such as the NCCPC-PV (Non-Communicating Children's Pain Checklist-Postoperative Version) and the modified FLACC scale have been validated for this population.

Clinicians should be mindful of the limitations inherent in using any scale, including the impact of sedative agents (e.g., ketamine, α2-agonists) on pain scores (20) and the necessity to apply age-specific ranges for each tool (9, 10, 21). Therefore, healthcare institutions should implement clear and simple local protocols to ensure the consistent and appropriate use of these assessment tools (5).

### Basic pharmacological and non-pharmacological pain management

This section outlines fundamental measures for preventing and treating postoperative pain. These include: the co-administration of paracetamol (acetaminophen) and non-steroidal anti-inflammatory drugs (NSAIDs, where not contraindicated), preoperative dexamethasone, and distraction/psychological interventions to reduce perioperative anxiety.

A basic treatment regimen should incorporate both paracetamol and NSAIDs (4, 22). It is important to note that paracetamol is contraindicated in patients with liver disease, while NSAIDs are contraindicated in patients with hypovolemia, renal impairment, and active infection. The optimal analgesic effect of paracetamol and NSAIDs is obtained when administered systematically during the first few days after surgery (22, 23). Research has demonstrated that this combination is effective in reducing postoperative pain and the requirement for rescue opioids in various surgeries (24, 25). Parental administration of opioids at home can be difficult and, if mismanaged, poses a risk of dependence and misuse, as documented internationally (26–28).

Dexamethasone, a potent steroid, used for its anti-emetic properties, offering substantial and prolonged pain relief (up to 48 h) (29) and an opioid-sparing effect (29, 30).

Because of its modulatory effect on the immune system, there is a concern about the occurrence of postoperative infections following the use of steroids. However, a large controlled trial involving over 8000 adult patients conducted by Corcoran et al. (31) found no evidence of an increased incidence of surgical site infections associated with the perioperative administration of 8 mg of dexamethasone (0.1 mg·kg^−^^1^).

In children studies, no correlation was found between dexamethasone administration and postoperative infectious complications (29, 30). Moreover, no dose-related relationship was identified between dexamethasone administration and the occurrence of postoperative infections following tonsillectomy (30). Although, the majority of studies conducted on this subject in children were designed to explore pain outcomes as the primary outcome (and thus may be subject to a selective bias), the extant evidence appears to indicate that dexamethasone is unlikely to be associated with surgical-site infection.

A further concern is the occurrence of postoperative bleeding associated with a dose-related dexamethasone administration in children after tonsillectomy (32). However, a review of high-quality studies, including the publication conducted by Gallagher et al. in adults (33) and more recent systematic meta-analyses exploring this topic in children (29, 30), has revealed no association between dexamethasone administration (even at 0.5 mg·kg^−^^1^) and serious bleeding complications, even when adjusting for dexamethasone dosage (30). A debate continues regarding the optimal dosage for achieving the desired level of analgesia (0.1 to 0.5 mg·kg^−^^1^). However, it is typically administered at its minimal effective dose (0.15 mg·kg^−^^1^ at anesthesia induction) (29, 34) to prevent the aforementioned complications.

While some practitioners use other steroids such as prednisolone for pain management after surgery, a recent meta-analysis found it ineffective for post-tonsillectomy pain (4, 35).

Possibly, most underutilized, are distractive and psychological therapies (36). Preoperative educational workshops designed for both children and their parents have been extensively investigated and demonstrated to effectively reduce pain and anxiety (36).

A recent meta-analysis examining additional modalities and techniques such as virtual reality, electronic games, and interactive robots device, indicates that they are also effective adjuncts for pain and anxiety management (36–38).

While the optimal technique(s) remain to be defined, caregivers should focus on two elements: providing a clear and comprehensive explanation of the perioperative process and offering reassurance to both children and their parents regarding potential sources of misunderstanding (39–41).

Furthermore, restricting the preoperative fasting interval to one hour prior to surgery, coupled with the prompt resumption of oral fluid intake postoperatively, has been shown to enhance the quality of pain management and reduce the need for opioid rescue analgesia (42, 43).

### Surgical techniques and enhanced recovery after surgery

Over the past decade, one of the most significant advancements in pain management has been the development and implementation of less invasive surgical techniques. This progression initially emerged with the widespread adoption of laparoscopic procedures for abdominal surgery (44, 45), which have now largely replaced traditional open surgeries for almost all relevant indications.

This shift has been associated with a reduction in postoperative pain, simplified pain management, and an overall enhancement of postoperative recovery (44). Recently, various surgical specialties have adopted similar minimally invasive methodologies. For example, in ENT surgery, techniques such as radiofrequency and coblation, frequently paired with partial tonsillectomy, have resulted in diminished postoperative pain, accelerated patient recovery—including a quicker resumption of a regular diet—and fewer complications such as bleeding (46).

In recent years, less invasive techniques have also been developed for major orthopedic surgeries. Published studies on percutaneous spinal surgery for scoliosis correction (47). While the specific advantages for pain management are still under investigation, a favorable impact is expected, given that postoperative pain has consistently been shown to correlate with the degree of surgical invasiveness. Given this proof, less invasive surgical techniques should be prioritized whenever feasible.

Surgical technique advancements have significantly improved postoperative pain management, but this represent only one component of the Enhanced Recovery After Surgery (ERAS) protocol (48, 49). ERAS is generally considered the most significant development in perioperative care over the past 20 years. The program encompasses a comprehensive set of preoperative, intraoperative, and postoperative measures designed to improve patient outcomes. ERAS protocols involve several key components: preoperative patient information, preoperative optimization of illness conditions, avoidance of preoperative bowel preparation, minimization of preoperative fasting with regular carbohydrate administration, avoidance of sedative premedication, antimicrobial prophylaxis; intraoperative use of fast-acting agents, extensive use of regional analgesia and reversal of muscle relaxation, minimal invasive surgery, maintenance of normothermia, adequate fluid management and avoidance of surgical drainage; and postoperatively: avoiding nasogastric tubes, removing all drainage as soon as possible, postoperative analgesia, nausea and vomiting prophylaxis, early oral feeding and early mobilization. A more detailed protocol can be assessed in more extensive review dedicated to pediatric ERAS (48–50). These protocols were modified for the pediatric population (see Table 2) (50, 51).

**Table 2:** ERAS components in pediatrics.

Preoperative period	Intraoperative period	Postoperative period
• Preoperative multimodal pain plan • Coaching and Expectation Building • Optimize medical comorbidities • Avoid prolonged preoperative fasting • Administer non-opioid analgesia and treatment of preoperative pain	• Venous thromboembolism prophylaxis • Pre-incision antibiotic prophylaxis • Standard anesthetic protocol • Regional anesthesia and short acting anesthetic • Minimally invasive technique • Prevention of nausea and vomiting • No nasogastric tubes • Standardized hypothermia prevention	• No intraperitoneal perianastomotic drains • Goal directed/near-zero fluid therapy • Early removal of urinary catheters • Prevention of postoperative ileus • Opioid-sparing pain regimen • Perioperative nutritional screening • Early mobilization • Audit protocol compliance and outcomes

While ERAS is often distinguished from ambulatory surgery, the two approaches should be considered as complementary, as ERAS principles promote the transition from inpatient to outpatient care. To date, numerous studies and systematic reviews have demonstrated that ERAS protocols in pediatric populations shorten hospital stays, lower complication rates, and improve postoperative pain management (49). Although pediatric-specific evidence continues to evolve, reduced postoperative morphine consumption has been identified as a crucial determinant in shortening hospital stays within ERAS frameworks (52). This emphasizes the vital need of limiting opioid use in the management of postoperative pain.

### Opioids, hyperalgesia and persistent pain after surgery

#### Opioids and related compounds

Opioids are considered the primary treatment for moderate to severe postoperative pain. The most common agents used are pure µ-opioid receptor agonists (e.g., morphine) and mixed agonist-antagonists (e.g., nalbuphine).

The primary advantage of using mixed agonist-antagonists is their ceiling effect on respiratory depression, which reduces the risk of severe complications (1). Nalbuphine is commonly used in Europe, typically as an initial bolus of 0.2 mg·kg^−^^1^ followed by a continuous infusion of 1 mg·kg^−^^1^·day^−^^1^. Its primary limitation is the aforementioned ceiling effect, which can result in inadequate analgesia for severe pain, necessitating a switch to a pure agonist. One theoretical concern is that its µ-receptor antagonism could reduce the efficacy of subsequent rescue doses of pure agonists.

Morphine, is the most common pure µ-agonist (1, 53). Recent recommendations has been published regarding the perioperative use of opioids in children (53, 54). Its pharmacokinetics in children are similar to adults, except in neonates, in whom dose adjustments are necessary due to hepatic immaturity (55–57). It is typically administered via patient-controlled analgesia (PCA) or nurse-controlled analgesia (NCA), with a specific target pain-scale score, underscoring the importance role of assessment.

Otherwise, morphine can also be administered as needed bolus dose (which is similar to NCA). A common practice in pediatrics is the use of a background infusion to maintain analgesia and sleep quality overnight, without increasing side effects. However, evidence indicates that such background infusions do not enhance postoperative pain control (58). Moreover, the continuous administration of opioids during pain-free intervals may potentially lead to an increase in both hyperalgesia and opioid consumption (58). Irrespective of the delivery method, routine monitoring of effectiveness and vital signs is imperative, along with established protocols for managing respiratory depression.

In the context of Enhanced Recovery After Surgery (ERAS) protocols has led to a shift from intravenous to oral morphine at the earliest opportunity to facilitate the removal of IV lines, although maintaining IV access enables prompt administration of naloxone if necessary (48).

Tramadol, another opioid analgesic, targets µ-, κ-, and δ-opioid receptors and inhibits norepinephrine and serotonin reuptake (23). It is metabolized by CYP2D6 into O-demethyltramadol (M1), which is 20 times more powerful than morphine. Similar to codeine, interindividual variability in CYP2D6 activity leads to unpredictable efficacy and risk (23). Standard dosing is 2 mg·kg^−^^1^ up to four times daily. Due to the risk of respiratory depression in patients with obstructive sleep apnea (increased opioid sensitivity) or who are CYP2D6 hypermetabolizers (59), it is strongly recommended to: 1) ensure that basic analgesic treatments (previously described) are in effect, and 2) test patient sensitivity with an in-hospital dose while monitoring respiratory function for two hours (the peak period for M1 effect) before discharge.

Notably, tramadol is strongly not recommended for use in children in many countries, including the United States (53, 54, 60).

Alternative to tramadol and codein are intravenous and oral opioids. Interestingly, the systematic administration of paracetamol and NSAIDs has been shown to increase the quality of analgesia while avoiding any administration of rescue opioids (22). Consequently, this association should be considered whatever possible.

#### Persistent pain after surgery and perspectives in its prevention

Despite its demonstrated effectiveness, there is an increasing tendency to restrict the use of morphine during and after surgery, partly due to concerns regarding opioid-induced hyperalgesia (OIH) (60–63). Although no causal link has been proven between perioperative opioid administration and the development of persistent of chronic pain after surgery, but a statistical connection between these two factors has been confirmed (6, 64, 65). In addition, a recent study of adults demonstrated that using nociceptive monitoring (the analgesia nociceptive index) to guide intraoperative opioid administration reduced postoperative consumption, highlighting the importance of prudent opioid use in preventing OIH (66).

Additionally, a recent meta-analysis conducted in adult populations found that the use of regional analgesia decreased the incidence of persistent pain at both 3 and 6 months post-surgery (67). Given that the same meta-analysis found regional analgesia leads to a reduction in opioid use, it can be hypothesized that a decrease in of early postoperative pain (responsible of hyperalgesia) and opioid analgesia (associated with OIH) might prevent persistent or chronic pain after surgery. This hypothesis is strongly supported by recent meta-analyses that identified a statistical relationship between an increased incidence of chronic postsurgical pain and both postoperative pain intensity and opioid consumption after surgery (68, 69).

Opioid-induced hyperalgesia (OIH), in conjunction with the extent of surgical intervention and opioid consumption, is hypothesized to contribute to the development of persistent (>3 months) and chronic (>6 months) neuropathic postsurgical pain (64, 68).

Chronic pain affects approximately 10% of pediatric surgical patients, with rates reaching 40%–50% following major procedures such as scoliosis correction (6). Other risk factors include female gender and the presence of early neuropathic pain, though these are not modifiable. Regarding the statistical association between opioid and Elements of ERAS reducing opioid consumption may offer protective benefits, though further evidence is required. Other proposed preventive strategies encompass the use anti-hyperalgesic agents, perioperative *α*2-agonists, and regional anesthesia (70). It is crucial to ensure that any reduction in morphine consumption does not compromise the quality of pain management, given the correlation between early pain intensity and chronic postsurgical pain (69).

##### Anti-hyperalgesic compounds

Ketamine, an NMDA receptor antagonist, is the most-well known agent in this class. However, its efficacy in children are less compelling; a recent meta-analysis demonstrated benefits primarily when ketamine was employed as an adjunct to regional anesthesia or within opioid-free analgesic protocols (71). Esketamine, a ketamine enantiomer, has been introduced more recently. However, despite the encouraging results with related to pain management (72), there remains a lack of data concerning the opioid-sparing effect of this compound. Gabapentin has shown promise in reducing pain and morphine consumption after pediatric spine surgery (73), yet a recent adult's meta-analysis found no preventive effect against chronic postsurgical pain (74). Considering the limited evidence for anti-hyperalgesic effects in children, a significant preventive effect appears improbable.

Finally, the most promising agent identified is Lidocaine. Two recent meta-analyses have found this compound to decrease postoperative opioid consumption in children, especially when it is administered intraoperatively and postoperatively, up to 6 h after surgery (longer durations have not been investigated). The standard dosage range is 1 to 1.5 mg.kg^−1^.h^−1^. However, the effects of prolonged postoperative administration have not been investigated (70, 75). The results of this meta-analysis have been disputed by a controlled study involving children undergoing scoliosis surgery (76). This study reported no opioid-sparing effect from perioperative lidocaine administration. If utilized, it seems prudent to recommend the administration of lidocaine during the initial 24 h post-surgery, as this duration has been identified as optimal in adult (77).

Methadone, a long-acting opioid with an antagonistic effect on the NMDA receptor, has attracted considerable interest. Research has demonstrated its efficacy in reducing opioid tolerance and OIH (78). A recent meta-analysis has summarized the results of the 5 available controlled trials. While the administration of intraoperative (0.1 to 0.2 mg.kg^−1^) prior to incision has been shown to reduce opioid consumption and enhance pain quality, the overall level of recommendation remains low due to the limited number of studies and methodological concerns associated with the included studies.

Similarly, magnesium sulfate, another NMDA receptor antagonist, has demonstrated potential in reducing the need for opioid rescue analgesia (79). However, the limited number of studies available precludes definitive recommendations regarding its clinical application.

##### Alpha 2 agonists—clonidine and dexmedetomidine

Clonidine offers both sedation and analgesia. It is frequently employed for extending the duration of regional blocks. Research indicates that its application as a premedication can lead to a reduction in postoperative pain (80, 81). Dexmedetomidine is used for sedation purposes and as an anesthetic adjuvant. A recent meta-analysis revealed that its use during surgery, in conjunction with general anesthesia, significantly lowers postoperative opioid consumption in pediatric patients (82). The majority of studies included in this meta-analysis typically administered an initial bolus of 1 µg.kg^−1^ followed by a continuous infusion of 0.4 µg.kg^−1^.h^−1^ (82).

##### Nociceptive monitoring

Evidence suggests that hyperalgesia may develop, at least in part, due to inappropriate use of opioids in the absence of nociceptive stimulation. This phenomenon has been extensively documented by Upton et al. (66) who observed a different profile in the administration of fentanyl during surgery, noting no significant changes in the total amount of intraoperative fentanyl given to both the ANI and control groups during anesthesia for spinal surgery in adults.

Furthermore, a recent large-scale study demonstrated that increased intraoperative fentanyl administration was associated with improved postoperative pain control and a reduction in early postoperative opioid consumption (83).

Furthermore, a recent large-scale study demonstrated that increased intraoperative fentanyl administration was associated with improved postoperative pain control and a reduction in early postoperative opioid consumption

This correlation has been attributed to enhanced management of intraoperative nociceptive stimulation, which facilitates better pain control in the postoperative phase (83, 84).

Almost all nociceptive monitoring techniques have been studied and validated in pediatric patients. These include pupillometry (85–88), analgesia nociception index (17, 88, 89) and nociception level index (16, 90, 91).

The majority of these devices have been shown to lead to a decrease in intraoperative opioid administration when they used to guide analgesia during the intraoperative period (92, 93).

Despite the absence of compelling evidence at this time (68, 69), it is conceivable that this would lead to a reduction in hyperalgesia and decrease the risk of persistent and chronic pain following surgery (62, 83).

##### Regional anesthesia and analgesia

Regional anesthesia/analgesia is a cornerstone of multimodal analgesia, aiming to reduce postoperative pain and decreasing intra- and postoperative opioid consumption. In many cases, RA is incorporated into enhanced recovery after surgery (ERAS) protocols, as it can support various aspects of patient care, such as earlier resumption of oral intake [e.g., cleft lip repair surgery (94)] or earlier mobilization [e.g., posterior spinal fusion surgery (95)].

Less frequently, regional analgesia is used in children for primary anesthetic purpose, such as spinal anesthesia for infra-umbilical surgery in neonates or limb surgery in adolescents. Table 3 illustrates recommended regional anesthesia techniques for pediatric-specific surgical procedures.

**Table 3:** Suggested regional anesthesia techniques for pediatric-specific surgical procedures.

Region	Surgery (examples)	Block	Regional anesthesia
Cephalic	Maxillofacial and labial surgery (cleft lip/palate repair, Lefort surgery)	Maxillary nerve block (V2) suprazygomatic approach	Ultrasound-guided
	Craniotomies (craniosynostosis, tumor resection)	Scalp block	Ultrasound-guided
Thorax	Pediatric thoracotomy	Thoracic epidural catheter	Landmark, ultrasound if difficulty anticipated
	Neonatal thoracotomy	Thoracic wall catheter	Surgical placement
	Pediatric thoracoscopy	Paravertebral block	Ultrasound-guided
	Neonatal thoracoscopy	Thoracic wall catheter	Surgical placement
Spine	Posterior spinal fusion	Intrathecal Morphine	Early intraoperative surgical injection
Abdomen	Deep visceral surgery (digestive resections, transanal pull-through)	Lumbar epidural catheter	Landmark, ultrasound if difficulty anticipated
	Superficial visceral surgery via laparotomy	Transversus abdominis plane (TAP) block	Ultrasound-guided
	Urologic surgery via lombotomy (pyeloplasty)	Quadratus lumborum (QL) block	Ultrasound-guided
	Laparoscopic surgery (cholecystectomy, appendectomy)	Trocar-site infiltration or TAP block	Ultrasound-guided
Pelvis	Pelvic urologic surgery (vesicoureteral reflux surgery)	Caudal epidural block	Ultrasound guidance desirable
	Circumcision	Bilateral pudendal nerve block Bilateral penile block	Nerve stimulation Ultrasound guidance
	Hypospadias repair	Bilateral pudendal nerve block Caudal epidural block	Nerve stimulation Ultrasound guidance desirable
	Cryptorchidism, orchidopexy	Ilioinguinal–iliohypogastric + pudendal block Caudal epidural block	Nerve stimulation Ultrasound guidance desirable
Lower limb	Unilateral hip or femur osteotomy	Erector Spine Block	Ultrasound-guided
	Bilateral hip or femur osteotomy	Lumbar epidural catheter	Ultrasound-guided

Since 2022, an international framework has been available to address the practical aspects of regional analgesia procedures (96). This development mirrors the widespread adoption of ultrasound guidance over the past fifteen years, facilitating the adoption of numerous peripherals, particularly interfascial blocks, for which no satisfactory landmark-based approach existed (97, 98). This, in turn, has contributed to the expansion of ambulatory surgery and to a reduction in the use of central neuraxial blocks, which are associated with an increased risk of complications (99). In pediatric medical practice, regional anesthesia procedures are performed under general anesthesia in the majority of cases. Consequently, early clinical indicators of complications may be more challenging to identify. This idea finds some support in the literature, as demonstrated by a 2021 review by Ramesh et al., which compiled 34 case reports of local anesthetic systemic toxicity in children, of which only four patients had benefited from ultrasound-guided approaches (100).

Nevertheless, the existing data provide reassurance concerning the safety of pediatric regional analgesia (101). Two large cohort studies demonstrate that complications are rare and that severe incidents are exceptional when best-practice recommendations are followed (102, 103).

The incidence of adverse events may even be lower under general anesthesia, likely due to the limited cooperation of awake children. This view is further supported by recent meta-analyses comparing ultrasound guidance with landmark-based techniques for central neuraxial blocks and specific peripheral procedures, which reported no instances of severe local anesthetic systemic toxicity. From an efficacy standpoint, these studies consistently demonstrate a benefit of ultrasound guidance, evidenced by lower failure rates and reduced need for postoperative rescue analgesia when ultrasound is used (104, 105).

Over the last ten years, there has been little change in the selection of local anesthetic agents.

Long-acting amide local anesthetics, such as the pure-S-enantiomers ropivacaine and levobupivacaine, offer the most favorable safety profile, particularly with regard to cardiotoxicity. Pediatric protocols generally recommend the use of low concentrations in order to promote a differential block targeting small-diameter fibers (A-delta and C fibers) involved in nociception. Higher volumes have been shown to improve longitudinal nerve coverage and signal suppression without reaching toxic thresholds (∼3 mg.kg^−1^) in patients with lower body weight (106).

Concentrations of around 2 mg.mL^−1^ are therefore the preferred as first-line options, with further dilutions to 1 or even 0.5 mg.mL^−1^ being considered for smaller children. However, the duration of action of these local anesthetics remains limited (approximately 6 h) and is often insufficient to cover the entire postoperative pain period, which may last several days following major surgery.

One approach to overcome his limitation is the placement of a perineural catheter, which allows for the continuous administration of local anesthetics over several days. Their efficacy has been relatively well established since 2010, particularly in major orthopedic surgery (101, 107–109).

Since then, little additional evidence has emerged, except for a longitudinal study published in 2014 confirming the feasibility of ambulatory use, provided that a well-structured care network is in place (110, 111). However, It should be noted that associated morbidity is not negligible: in a 2015 study involving nearly 2,100 perineural catheters, complications were reported in approximately 10% of patients (112). The majority of these issues were mechanical in nature and resulted in catheter dysfunction. Infections, which occurred in nearly 1% of patients, were significantly more frequent when the catheter duration exceeded three days, supporting recommendations not to exceed this time frame. The safety of continuous blocks primarily depends on organizational protocols including follow-up and monitoring of pain intensity and skin, caregiver education, and the availability of anesthesiologist (113).

Another commonly used strategy to enhance and prolong analgesia is the administration of adjuvant agents. These adjuvants can be administered either intravenously or via the regional route by adding them to the local anesthetic solution.

Dexamethasone, which has previously been noted for its intrinsic antiemetic and opioid-sparing properties, also prolongs the duration of analgesic blocks. Due to preclinical data suggesting the possibility of neurotoxicity with perineural administration, intravenous administration is generally preferred (114). The most extensively studied indication in the literature is caudal anesthesia, with three meta-analyses showing consistent efficacy findings without significant differences in infectious or metabolic complications (115–117). However, dosing regimens are not standardized, with reported doses ranging from 0.1 to 0.5 mg.kg^−1^.

Alpha-2 adrenergic agonists represent the second most frequently used class of adjuvants, with a high level of evidence supporting their efficacy of both dexmedetomidine (118, 119) and clonidine (120, 121). From a safety perspective, the data on clonidine is well-established and longstanding, while some studies report a slightly increased risk of bradycardia associated with dexmedetomidine (122). Regarding dosing, the literature tends to agree on a dose of 1 µg.kg^−1^, which provides satisfactory efficacy without increasing sedation or cardiovascular risk.

Although there is a wealth of evidence concerning acute pain, data on chronic pain outcomes remains considerably limited. This is a significant concern, as chronic postoperative pain affects almost one in five patients (123).

Nowadays, no studies have demonstrated a direct effect of regional anesthesia on chronic postoperative pain. However, indirect evidence support this hypothesis, as the intensity of acute postoperative pain is a major risk factor for pain chronicization (68).

### Organization, staffing and education

A concise summary of this section would be as follows in two words: multidisciplinary work.

Indeed, it is widely demonstrated in the literature that cooperation and mutual understanding enhance patient outcomes and care coordination (124–127).

Furthermore, the increasingly complex nature of surgical pathways, which involve numerous medical and paramedical professionals, alongside the introduction of ERAS protocols, which are inherently multidisciplinary (48), necessitates that care relies on meticulous coordination among the various parties involved. Pain management is not exempt from this logic. Developing agreed-upon protocols among professionals and explaining them to all stakeholders involved in pain management is therefore appropriate (113, 128).

Additionally, continuous professional education is essential, especially for nurses involved in administering opioids and managing regional analgesia catheters (128). Anesthesiologists also need to receive training in advanced regional techniques, including the use of ultrasound guidance.

Finally, due to patient variability and the wide age range in pediatrics, which affects analgesic response, a specialized team is essential to ensure not only the effective short-term pain management but also for monitoring patients experiencing persistent post-surgical pain.

### Practical management of pain

Strong evidence supports many of these points. Postoperative pain management should begin before surgery by alleviating anxiety using non-pharmacological interventions, such as preoperative workshops, videos or distractions. Clonidine premedication can be considered due to its effectiveness in managing postoperative pain. Postoperatively, the recommended primary treatment approach for patients should involve the use of non-opioid analgesics, such as paracetamol, NSAIDs, and low-dose dexamethasone.

Regional anesthesia should be performed whenever possible. Although opioids are recommended for major surgery or if regional anesthesia is incomplete or absent, an opioid-sparing strategy should be employed using intraoperative intravenous dexmedetomidine (either as a bolus or as a bolus followed by a continuous administration for long surgical procedures).

Postoperative pain management should align with ERAS protocols when inpatient care is required. Finally, educating caregivers and parents is crucial, particularly regarding opioid administration, monitoring, and the risk of chronic misuse. Although various alternatives such as nociception monitoring, methadone, lidocaine, or esketamine are available, the limited literature does not support any definitive recommendations for their use.

Readers can find as an open-assess recent recommendations concerning the management of different surgical conditions in the following two reviews: Postoperative pain management in children: Guidance from the pain committee of the European Society for Paediatric Anaesthesiology (ESPA Pain Management Ladder Initiative) (5, 129). Additionally, specific recommendations have also been developed for appendectomy (130).

## Conclusion

Although the fundamental principles of postoperative pain management have remained largely consistent, significant developments have occurred over the past decade. One of the primary catalysts has been the widespread adoption of Enhanced Recovery After Surgery (ERAS) protocols in pediatric populations. ERAS has transformed postoperative recovery pathways and fundamentally influenced pain management strategies. A central tenet of ERAS is the deliberate reduction of postoperative opioid use, which has directly altered prescribing practices, including those for morphine.

A second, critical evolution is the growing recognition of chronic postsurgical pain as a significant pediatric patient outcome. Evidence now underscores a statistical relationship between the quality of acute postoperative pain management—encompassing both pain intensity and opioid exposure—and the incidence of this long-term complication. In response, the strategic reduction of opioids, coupled with the increased use of regional anesthesia techniques, has become a primary goal in modern postoperative pain management plans.
